# SMARCA4缺失型肺部肿瘤放疗疗效及相关参数分析

**DOI:** 10.3779/j.issn.1009-3419.2026.102.15

**Published:** 2026-05-20

**Authors:** Yu GU, Ruxi GAO, Xingwen FAN, Xiaoxiao GE

**Affiliations:** 1 200032 上海，复旦大学附属肿瘤医院放疗科; 1 Department of Radiation Oncology, Fudan University Shanghai Cancer Center, Shanghai 200032, China; 2 200032 上海，复旦大学上海医学院肿瘤学系; 2 Department of Oncology, Shanghai Medical College, Fudan University, Shanghai 200032, China; 3 200032 上海，复旦大学附属中山医院临床医学研究院; 3 Clinical Research Center, Zhongshan Hospital Fudan University, Shanghai 200032, China

**Keywords:** 肺肿瘤, SMARCA4缺失, 未分化肿瘤, 放射治疗, 免疫治疗, Lung neoplasms, SMARCA4 deficiency, Undifferentiated tumor, Radiotherapy, Immunotherapy

## Abstract

**背景与目的** SMARCA4缺失型肺部肿瘤是近年来肺癌研究的热点领域之一，该亚型侵袭性强、预后差，但其放疗相关研究仍十分匮乏，放疗参数的选择及联合免疫治疗的时序缺乏循证依据。本研究评估放疗在该类肿瘤中的疗效，并探讨放疗参数的预后价值。 **方法** 回顾性分析88例SMARCA4缺失型肺部肿瘤患者，分为放疗组（*n*=20）和未放疗组（*n*=68）。采用*Kaplan-Meier*法比较组间总生存（overall survival, OS），并分析放疗部位、生物等效剂量（biological effective dose, BED）、分割模式及免疫治疗时序对预后的影响。**结果** 放疗组年龄≥60岁比例更低（25.0% *vs* 66.2%,* P*<0.001），接受免疫治疗比例更高（65.0% *vs* 33.8%, *P*=0.013）。放疗组中位OS未达到，显著优于未放疗组（22.9个月，*P*=0.048）；放疗人群中，放疗联合免疫治疗组中位OS显著优于单纯放疗组。放疗部位、BED、分割方式和治疗时序组间的OS差异均无统计学意义。**结论** 放疗是改善SMARCA4缺失型肺部肿瘤患者OS的有效手段，联合免疫治疗可协同增效；放疗参数对预后无显著影响，有利于临床方案简化；放疗与免疫治疗时序需要优化，当前探索性结论为后续大样本研究提供了重要的假设基础。

## Abstract (en)

**Background and objective** SMARCA4-deficient pulmonary neoplasms have emerged as a major focus of lung cancer research in recent years. This subtype is characterized by aggressive behavior and poor prognosis. However, radiotherapy-related studies in this population remain scarce, and evidence is lacking regarding the optimal selection of radiotherapy parameters and the sequencing of combination with immunotherapy. The present study aimed to evaluate the efficacy of radiotherapy in these tumors and to explore the prognostic value of radiotherapy parameters. **Methods** Clinical data of 88 patients with SMARCA4-deficient pulmonary neoplasms were retrospectively analyzed. Patients were divided into a radiotherapy group (*n*=20) and a non-radiotherapy group (*n*=68). Overall survival (OS) was compared between groups using the *Kaplan-Meier* method. Subgroup analyses were performed for radiotherapy site, biological equivalent dose (BED), fractionation mode, and the timing of radiotherapy combined with immunotherapy. **Results** The radiotherapy group had a lower proportion of patients aged ≥60 years (25.0% *vs* 66.2%, *P*<0.001) and a higher proportion receiving immunotherapy (65.0% *vs* 33.8%, *P*=0.013) compared with the non-radiotherapy group. The median OS was not reached in the radiotherapy group, which was significantly superior to that in the non-radiotherapy group (22.9 mon, *P*=0.048). Among patients receiving radiotherapy, those who also received immunotherapy had a significantly longer median OS than those receiving radiotherapy alone. No statistically significant differences in OS were observed among subgroups stratified by radiotherapy site, BED, fractionation schedule or timing. **Conclusion** Radiotherapy effectively improves OS in SMARCA4-deficient lung tumors, with synergistic potential when combined with immunotherapy. The lack of prognostic impact of radiotherapy parameters supports regimen simplification, while the timing of combined therapy needs refinement. These exploratory results lay a critical foundation for future large-sample confirmatory studies.

SMARCA4（BRG1）是SWI/SNF染色质重塑复合体的核心ATP酶亚基，在转录调控及DNA损伤修复中发挥关键作用^[1]^。SMARCA4基因突变及蛋白表达缺失在多种恶性肿瘤中均有报道，与肿瘤侵袭性行为和不良预后密切相关^[2-4]^。在胸部肿瘤中，SMARCA4缺失可见于非小细胞肺癌（non-small cell lung cancer, NSCLC）和新近认识的SMARCA4缺失型未分化肿瘤（SMARCA4-deficient undifferentiated tumor, SD-UT）^[5]^。前者在NSCLC中突变率为5%-10%，具有更强的侵袭性和更差的预后；后者是2021年世界卫生组织（World Heanlth Organization, WHO）胸部肿瘤分类正式确认的独立实体，以未分化/横纹肌样形态及SMARCA4蛋白表达缺失为特征，临床上，该病好发于年轻男性，与重度吸烟密切相关，常表现为纵隔巨大侵袭性肿块，易侵犯周围器官，临床进展极为凶险，中位总生存期（overall survival, OS）短^[5-7]^。两类疾病总体罕见，目前国内外报道多为小样本病例系列。

目前，SMARCA4缺失型肺部肿瘤尚无标准治疗方案^[8]^。SMARCA4缺失型NSCLC通常参照NSCLC指南治疗，而SD-UT多采用铂类化疗或肉瘤方案，疗效有限。免疫检查点抑制剂（immune checkpoint inhibitors, ICIs）的应用存在争议^[9-11]^：部分研究提示SMARCA4缺失与高肿瘤突变负荷及较好的免疫治疗反应相关，亦有研究报道SD-UT以免疫沙漠型微环境为主，ICIs单药疗效有限。由于疾病罕见，治疗决策多基于小样本回顾性数据。

放疗在SMARCA4缺失型肺部肿瘤中的报道甚少^[12]^。临床前研究^[13]^提示，SMARCA4参与DNA双链断裂修复，其缺失可能增强肿瘤细胞放射敏感性，提示放疗可能具有特殊价值。然而，目前尚无研究对放疗参数（剂量、分割模式、靶区）及放疗-免疫时序进行细化分析。

基于上述背景，本研究回顾性分析88例SMARCA4缺失型肺部肿瘤患者的临床资料，旨在评估放疗对OS的影响，分析放疗联合免疫治疗的协同效应，并探索不同放疗参数及治疗时序对预后的影响，为临床实践提供参考。

## 1 资料与方法

### 1.1 临床资料

本研究回顾性收集2017年1月至2023年8月于复旦大学附属肿瘤医院经病理确诊的SMARCA4缺失型肺部肿瘤患者的临床资料。SMARCA4/BRG1抗体（克隆号EPNCIR111A，稀释度1:100；英国剑桥Abcam公司），肿瘤细胞中未出现明显的核染色现象，表明SMARCA4蛋白存在“缺失或表达不足”。以正常上皮细胞、炎症细胞及成纤维细胞的清晰核染色信号作为阳性内对照。最终纳入88例患者，其中SMARCA4缺失型NSCLC 64例，SD-UT 24例。

### 1.2 纳入与排除标准

纳入标准：（1）经病理确诊为SMARCA4缺失型肺部肿瘤（SMARCA4缺失型NSCLC或SD-UT）；（2）临床及随访资料完整；（3）至少接受过一线抗肿瘤治疗。排除标准：（1）合并其他恶性肿瘤；（2）临床资料严重缺失或失访。

### 1.3 资料收集

通过医院电子病历系统收集患者以下资料：（1）人口学特征：年龄、性别、吸烟史；（2）肿瘤特征：病理类型、肿瘤原发灶-淋巴结-转移（tumor-node-metastasis, TNM）分期[参照美国癌症联合委员会（American Joint Committee on Cancer, AJCC）第8版]、程序性细胞死亡配体1（programmed cell death ligand 1, PD-L1）肿瘤细胞阳性比例分数（tumor proportion score, TPS）；（3）治疗信息：是否接受放疗、放疗部位（原发灶/转移灶）、放疗分割方案、是否接受免疫治疗、免疫治疗药物、放疗与免疫治疗的时间间隔、是否接受手术、化疗及抗血管生成药物；（4）随访资料：生存状态及死亡时间。末次随访时间为2026年3月15日。

### 1.4 放疗参数分组

（1）放疗部位：原发灶放疗 vs 转移灶放疗。（2）生物等效剂量（biological equivalent dose, BED）：按公式BED=总剂量×（1+单次剂量/α/β）计算，其中α/β取10。以BED=70 Gy为界，分为BED≥70 Gy组和BED<70 Gy组。（3）分割模式：常规分割（1.8-2.0 Gy/次） vs 大分割（单次剂量≥3.0 Gy）。（4）放疗-免疫时序：同期联合（放疗与免疫治疗间隔≤14天） vs 序贯联合（间隔>14天）。

### 1.5 观察终点

主要观察终点为OS，定义为从确诊至任何原因死亡或末次随访的时间。次要观察终点为不同治疗模式及放疗参数亚组的OS差异。

### 1.6 统计学方法

采用SPSS 26.0统计软件进行数据分析。分类变量以例数（百分比）表示，组间比较采用卡方检验或Fisher精确检验。生存曲线采用Kaplan-Meier法绘制，组间比较采用Log-rank检验。所有检验均为双侧，P<0.05为差异有统计学意义。

## 2 结果

### 2.1 基线资料分析

2017年1月至2023年8月共纳入88例SMARCA4缺失型肺部肿瘤患者，其中放疗组20例，未放疗组68例。放疗组与未放疗组在性别、吸烟史、病理类型、TNM分期、PD-L1 TPS表达、手术及抗血管生成药物使用比例方面差异均无统计学意义（均P>0.05）。但放疗组年龄≥60岁患者比例显著低于未放疗组（25.0% vs 66.2%, P<0.001），且放疗组接受免疫治疗的比例显著高于未放疗组（65.0% vs 33.8%, P=0.013）（表1）。

**表1:** 患者基线临床特征

Characteristic	RT group (n=20)	Non-RT group (n=68)	χ^2^	P
Age (yrs)			10.680	<0.001
<60	15 (75.0%)	23 (33.8%)		
≥60	5 (25.0%)	45 (66.2%)		
Gender			0.867	0.405
Male	19 (95.0%）	67 (98.5%）		
Female	1 (5.0%)	1 (1.5%)		
Smoking history			0.196	0.482
Yes	16 (80.0%)	59 (86.8%)		
No	2 (10.0%)	5 (7.3%)		
Unknown	2 (10.0%)	4 (5.9%)		
Pathological type			0.067	0.520
NSCLC	15 (75.0%)	49 (72.1%)		
SD-UT	5 (25.0%)	19 (27.9%)		
TNM stage			0.612	0.301
I-III	11 (55.0%)	31 (45.6%)		
IV	8 (40.0%)	34 (50.0%)		
Unknown	1 (5.0%)	3 (4.4%)		
PD-L1 TPS			0.103	0.509
<1%	6 (30.0%)	12 (17.6%)		
≥1%	6 (30.0%)	15 (22.1%)		
Unknown	8 (40.0%)	41 (60.3%)		
Immunotherapy			6.214	0.013
Yes	13 (65.0%)	23 (33.8%)		
No	7 (35.0%)	45 (66.2%)		
Surgery			0.246	0.411
Yes	7 (35.0%)	28 (41.2%)		
No	13 (65.0%)	40 (58.8%)		
Anti-angiogenic therapy			0.041	0.546
Yes	3 (15.0%)	9 (13.2%)		
No	17 (85.0%)	59 (86.8%)		

RT: radiotherapy; NSCLC: non-small cell lung cancer; SD-UT: SMARCA4-deficient undifferentiated tumor; TNM: tumor-node-metastasis; PD-L1: programmed death ligand 1; TPS: tumor proportion score.

### 2.2 放疗对OS的影响

截至2026年3月，全组88例患者中位OS为25.5个月（0.7-98.8个月）。生存分析显示，未放疗组中位OS为22.9个月（0.7-98.8个月），4年OS率为30.8%；放疗组中位OS未达到（7.1-62.7个月），4年OS率为58.9%，两组间差异有统计学意义（Log-rank P=0.048）（图1）。

**图1:**
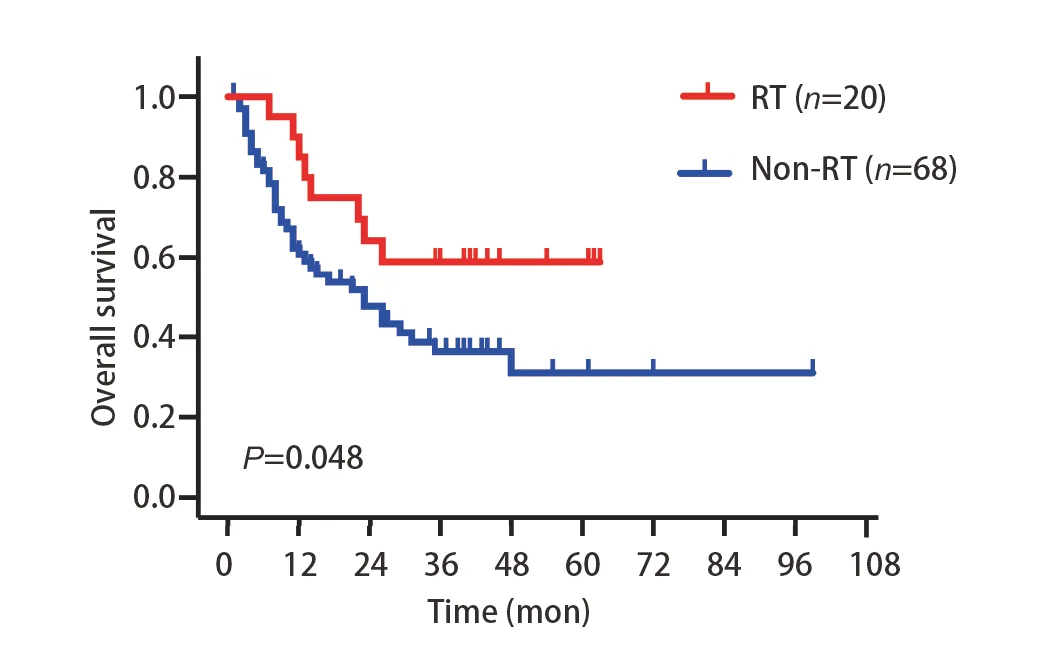
放疗组与未放疗组SMARCA4缺失型肺部肿瘤患者的OS曲线。采用Kaplan-Meier法绘制生存曲线，Log-rank检验进行组间比较。

### 2.3 放疗与免疫治疗的协同效应分析

为进一步评估放疗联合免疫治疗的协同效应，将接受放疗的患者按是否联合免疫治疗分为放疗+免疫组（n=13）和单纯放疗组（n=7）。结果（图2）显示：单纯放疗组中位OS为21.9个月（7.1-26.4个月），放疗+免疫组中位OS未达到（11.2-62.7个月），两组间差异有统计学意义（Log-rank P=0.001），提示放疗联合免疫治疗可显著延长患者生存，具有协同增效趋势。

**图2:**
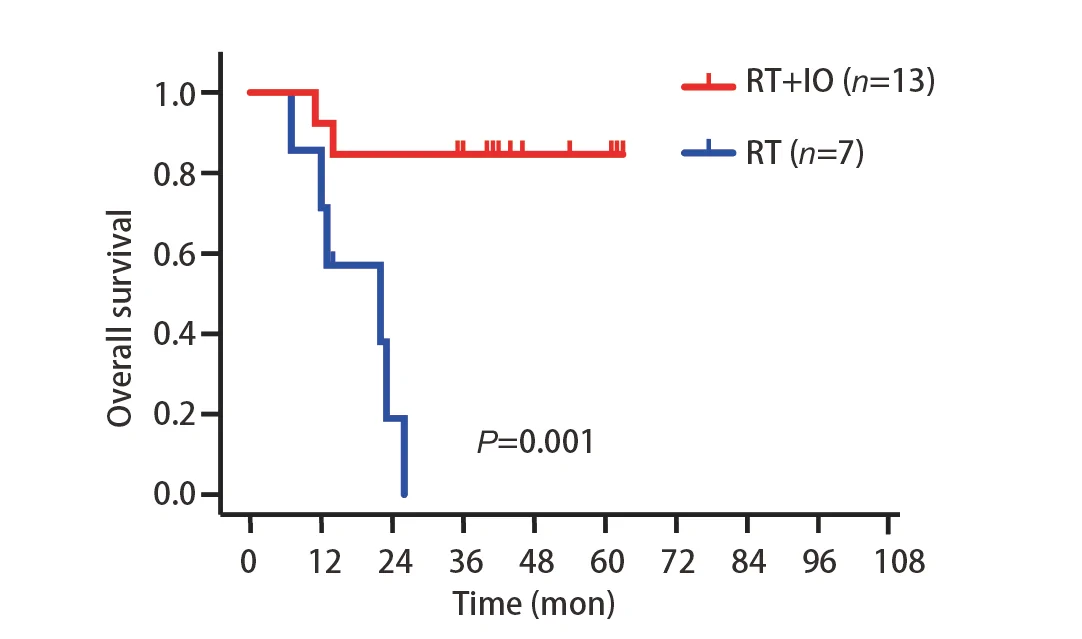
放疗联合免疫治疗组与单纯放疗组SMARCA4缺失型肺部肿瘤患者的OS曲线。采用Kaplan-Meier法绘制生存曲线，Log-rank检验进行组间比较。

### 2.4 放疗参数亚组分析

对20例接受放疗的患者进行亚组分析，结果如表2及图3显示：放疗部位（原发灶 vs 转移灶）、BED（≥70 Gy vs <70 Gy）及分割模式（常规分割 vs 大分割）组间的OS差异均无统计学意义（P均>0.05），提示SMARCA4缺失的肺部肿瘤对放疗具有普遍敏感性，且放疗联合免疫治疗带来的生存协同效应可能不依赖于具体的放疗技术参数。

**表2:** 放疗参数亚组的总生存比较

Radiotherapy parameter	n	2-yr OS rate	P
Radiotherapy site			0.429
Primary lesion	11	53.0%	
Metastatic lesion	9	77.8%	
BED level			0.617
<70 Gy	13	69.2%	
≥70 Gy	7	57.1%	
Fractionation mode			0.491
Conventional fractionation (1.8-2.0 Gy/Fx)	13	59.8%	
Hypofractionation (≥3 Gy/Fx)	7	71.4%	
RT-IO timing			0.173
Concurrent (interval ≤14 days)	7	71.4%	
Sequential (interval >14 days)	6	100.0%	

BED: biological equivalent dose; Fx: fraction.

**图3:**
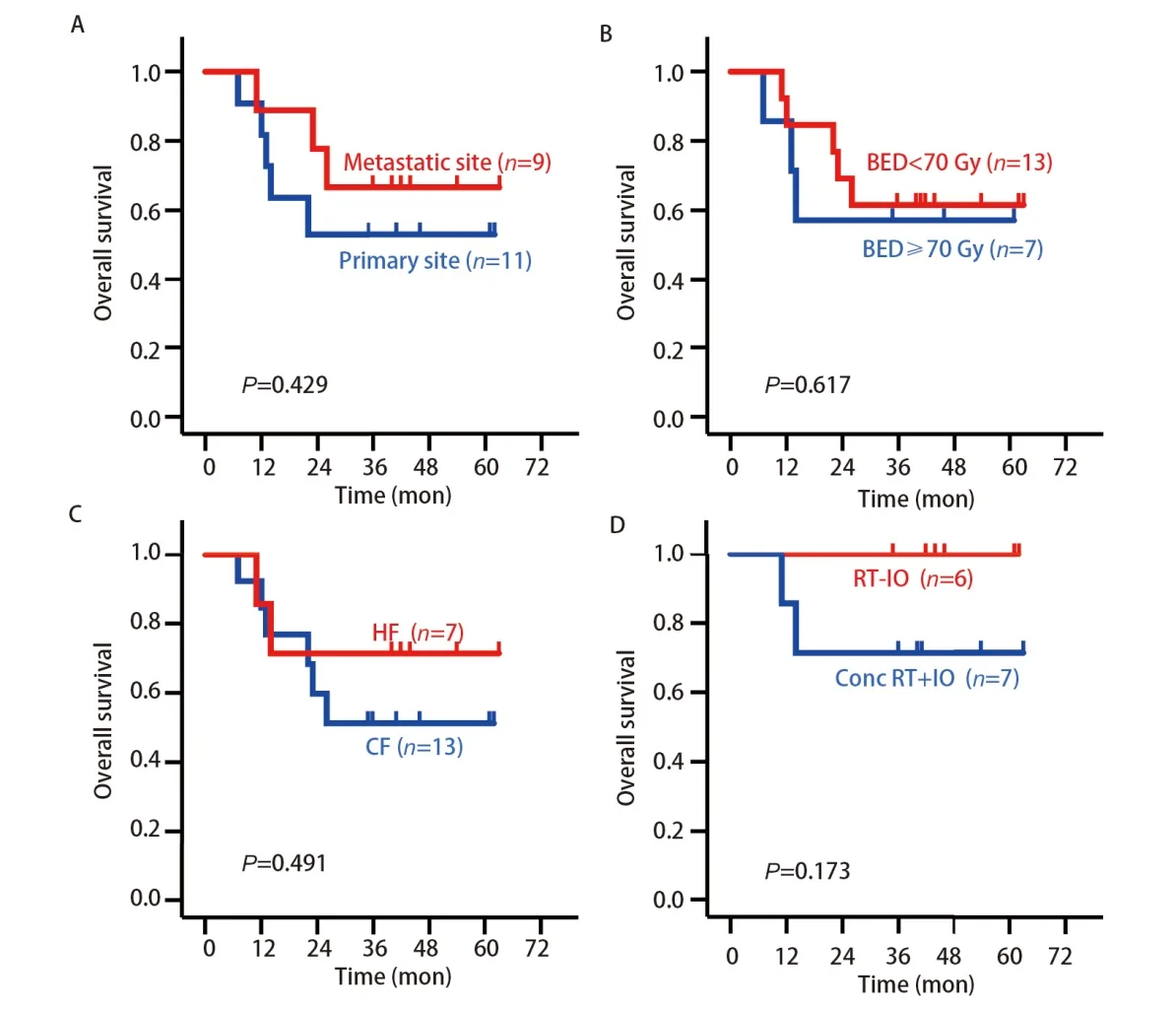
不同放疗参数亚组SMARCA4缺失型肺部肿瘤患者的总生存曲线。采用Kaplan-Meier法绘制生存曲线，Log-rank检验进行组间比较。A：放疗部位：原发灶 vs 转移灶；B：BED：≥70 Gy vs <70 Gy；C：分割模式：常规分割（CF）vs 大分割（HF）；D：放免联合时序：同期联合（Conc RT+IO）vs 序贯联合（RT-IO）。

### 2.5 放疗与免疫治疗时序分析

在13例接受放疗+免疫联合治疗的患者中，同期联合（间隔≤14天）者7例，2年OS率为71.4%，序贯联合（间隔>14天）者6例，2年OS率为100.0%；时序分析提示序贯治疗者的OS较同期联合者有延长趋势，但差异未达统计学意义（P=0.173）（表2，图3）。该趋势可能与样本量较小有关，有待进一步研究验证。

## 3 讨论

SMARCA4缺失型肺部肿瘤是一类罕见且高度恶性的胸部肿瘤，目前尚无标准治疗方案^[14]^。本研究基于单中心88例患者的真实世界数据，评估了放疗在SMARCA4缺失型肺部肿瘤中的疗效，并分析了不同放疗参数及放疗-免疫时序对预后的影响。

本研究发现，放疗组患者的中位OS显著优于未放疗组。但放疗组年龄≥60岁患者比例更低，接受免疫治疗的比例更高，反映了真实世界中的选择偏倚——年龄更轻、体能状态更好的患者更可能被推荐接受放疗和免疫治疗等积极干预措施^[15]^。尽管如此，放疗组中位OS较未放疗组延长的差距明显，难以仅由年龄和免疫治疗比例差异解释，提示放疗本身具有重要价值。

放疗联合免疫治疗显著优于单纯放疗组。放疗可通过诱导免疫原性细胞死亡、释放肿瘤相关抗原、激活T细胞应答，将“冷”肿瘤转化为“热”肿瘤^[16,17]^。SMARCA4缺失的肿瘤因染色质重塑异常导致基因组不稳定性增加，可能对放疗联合免疫治疗更为敏感^[18,19]^。

亚组分析显示，放疗部位（原发灶 vs 转移灶）、BED（≥70 Gy vs <70 Gy）及分割模式（常规分割 vs 大分割）对OS的影响均无统计学差异，提示SMARCA4缺失型肺部肿瘤可能对放疗具有普遍敏感性，且放疗诱导的远隔效应或免疫增敏作用可能不依赖于具体的放疗技术参数。SMARCA4直接参与DNA双链断裂修复，其缺失导致肿瘤细胞DNA修复能力受损^[20]^，因此即使常规分割、中等甚至低剂量放疗也可能达到理想效果。据此推测，对于无法耐受大分割或高剂量放疗的患者，适当降低放疗强度联合免疫治疗亦有可能带来生存获益。鉴于疾病罕见性受限，小样本下生存估计的精度有限，未来仍需更大样本量的研究以进一步验证。

放疗与免疫治疗时序方面，序贯联合者的OS较同期联合者呈延长趋势，但差异无统计学意义，可能与放疗后肿瘤抗原释放及T细胞启动需要一定时间窗口有关^[18,21]^。该趋势提示，放疗先行诱导免疫原性细胞死亡、释放肿瘤抗原，随后免疫治疗激活T细胞的时序策略可能具有一定的临床探索价值，但受限于两组样本量均较小，统计学效能不足，该假设有待进一步验证。

本研究为单中心回顾性设计，存在一定的局限性。首先，作为单中心回顾性分析，放疗组与未放疗组在年龄构成及免疫治疗接受比例上存在基线不均衡，这反映了真实世界临床实践中的决策倾向，属于回顾性研究的固有选择偏倚。此外，由于本研究中患者的放疗方案（包括靶区选择、剂量及分割方式）依据个体病情而定，存在一定的异质性。上述因素提示，在将本研究结论向更广泛的患者群体外推时，需审慎考虑基线特征差异对结果的影响。尽管如此，本研究作为目前已知较大样本的放疗疗效分析，仍为这一罕见肿瘤的临床决策提供了有价值的参考。基于上述发现，建议对SMARCA4缺失型肺部肿瘤患者在综合治疗中积极考虑放疗，尤其是联合免疫治疗；放疗参数选择可适当放宽，临床决策应更多基于患者耐受性。未来需多中心、前瞻性研究进一步验证。
